# Nanometer‐Scale 1D Negative Differential Resistance Channels in Van Der Waals Layers

**DOI:** 10.1002/advs.202408090

**Published:** 2024-11-13

**Authors:** Qirong Yao, Jae Whan Park, Choongjae Won, Sang‐Wook Cheong, Han Woong Yeom

**Affiliations:** ^1^ Center for Artificial Low Dimensional Electronic Systems Institute for Basic Science (IBS) Pohang 37673 South Korea; ^2^ Laboratory for Pohang Emergent Materials Department of Physics Pohang University of Science and Technology Pohang 37673 South Korea; ^3^ Max Plank Pohang University of Science and Technology (POSTECH) Center for Complex Phase Materials Pohang University of Science and Technology Pohang 37673 South Korea; ^4^ Rutgers Center for Emergent Materials and Department of Physics and Astronomy Rutgers University Piscataway NJ 08854‐8019 USA; ^5^ Department of Physics Pohang University of Science and Technology Pohang 37673 South Korea

**Keywords:** 1T‐TaS_2_, density‐functional calculations, interlayer orbital overlap, negative differential resistance, scanning tunneling microscopy and spectroscopy

## Abstract

Negative differential resistance (NDR) is the key feature of resonant tunneling diodes exploited for high‐frequency and low‐power devices and recent studies have focused on NDR in van der Waals heterostructures and nanoscale materials. Here, strong NDR confined along a 1‐nm‐wide 1D channel within a van der Waals layer 1T‐TaS_2_ is reported. Using scanning tunneling microscopy, a double 1D NDR channel formed along the sides of a charge–density‐wave domain wall of 1T‐TaS_2_ is found. The density functional theory calculation elucidates that the strong local band‐bending at the domain wall and the interlayer orbital overlap cooperate to bring about 1D NDR channels. Furthermore, the NDR is well controlled by changing the tunneling junction distance. This result would be important for nanoscale device applications based on strong nonlinear resistance within van der Waals material architectures.

## Graphical abstract

This work demonstrates 1D negative differential resistance (NDR) channels in a layered van der Waals material with charge density waves by using scanning tunneling microscopy and spectroscopy techniques. The mechanism of the NDR behavior is well elucidated. Moreover, the controllability of NDR in this system makes such nanometer‐scale 1D configuration a promising platform for logic nanodevice applications.

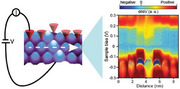

## 1 Introduction

Since the pioneering discovery by Esaki in a *p–n* junction of a germanium diode,^[^ ^1^ ^]^ negative differential resistance (NDR), defined by a decrease in current with increasing voltage, has been studied intensively in a wide range of device applications.^[^ ^2^, ^3^, ^4^, ^5^ ^]^ This nonlinear resistance behavior is the basis for a resonant tunneling diode, which has played an important role in high‐frequency and low‐power semiconductor devices. NDR behaviors have been observed in various materials not only with different dimensions from 0D to 3D but also with a widely varying size down to nanometer or molecular scale,^[^ ^6^, ^7^, ^8^, ^9^ ^]^ which has led to NDR research for nano or molecular electronics. However, it has been challenging to establish a well‐defined and robust nano or molecular scale material platform with a strong NDR behavior as well as to understand the atomic or molecular scale mechanism of NDR therein. More recently, a huge research interest has been built up in finding NDR behaviors in van der Waals atomic layers including graphene and transition metal dichalcogenides.^[^ ^10^, ^11^, ^12^ ^]^ This interest is partly fueled by the current interest in multi‐valued logic devices in 2D materials utilizing nonlinear resistivity. In these materials, well‐defined energy levels of a van der Waals atomic layer and versatile controllability over heterointerfaces could provide an ideal platform for NDR and its device architectures. While most of the previous works on NDR in van der Waals materials focused on vertical heterostructures, the nanoscale NDR embedded within a van der Waals layer has been rare in spite of its definite merit for 2D nanoscale device architectures.

Here, we introduce an NDR state confined along a well‐defined 1‐nm‐wide 1D channel within a single van der Waals layer of 1T‐TaS_2_. 1T‐TaS_2_ itself, a layered van der Waals material with charge–density‐wave (CDW) and an intriguing metal–insulator transition, possesses a giant potential for applications as nonvolatile multi‐valued memory devices with ultrafast switching speed.^[^ ^13^, ^14^ ^]^ Controlling its resistivity is crucial for such devices, which was demonstrated to be made possible by exciting various domain walls (DW) with temperature, light pulse, pressure, or doping in static or dynamic ways over the whole resistivity range spanning from an insulating phase to a metal phase and superconductivity.^[^ ^15^, ^16^ ^]^ Not limited to the in‐plane 2D resistivity, the out‐of‐plane resistivity could also be modulated by the interlayer orbital order.^[^ ^17^ ^]^ Namely, 1T‐TaS_2_ bulk or thin films have diverse stacking configurations, which host various insulating and metallic electronic states with different degrees of electron correlation.^[^ ^18^, ^19^ ^]^

Recently, very weak NDR of the 2D CDW superstructure was observed in its unoccupied state,^[^ ^20^ ^]^ which was related to the interplay of intralayer and interlayer coupling of the unoccupied Ta *d_z_* orbitals localized within each CDW unitcell.

In our present work, we discover strong 1D NDR channels of a 1‐nm‐scale width formed along a particular type of domain wall (DW) of the CDW in 1T‐TaS_2_. The 1D NDR channels are clearly visualized by mapping I/V curves using scanning tunneling microscopy (STM) to have a width of a single CDW unitcell of about 1 nm. The NDR channels appear in the occupied states at both sides of the DW symmetrically. The atomic structure and electronic states of the DW are determined through extensive density functional theory (DFT) calculations as guided by STM data. The calculations elucidate that the NDR is caused by indirect interlayer coupling of the occupied Ta *d_z_* orbitals localized within each CDW unitcell. Furthermore, the on/off of NDR is readily controlled via the external electric field induced by the STM tip, making the exploitation of this 1D NDR channel for van der Waals nanodevices promising.

## 2 Results and Discussion

### 2.1 1D NDR Channels on 1T‐TaS_2_ Surface

**Figure 1:**
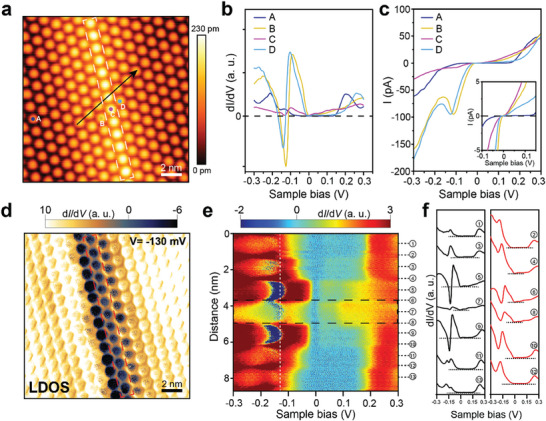
Observation of 1D NDR channels. a) STM topography image of 1T‐TaS_2_ surface with a bright chain‐like protrusion under a sample bias of +0.4 V. b) STS spectra obtained from the spots A–D in (a). The setting tunneling current is 100 pA. c) Corresponding I–V curves of the spots A–D. d) Map of local density of states for the surface in (a) under a scanning bias of −130 mV, obtained with a STM constant‐height mode. e) Line‐STS mapping along the black arrow in (a) from −0.3 to +0.3 V. f) Corresponding dI/dV curves taken at the positions in (e), odd number stands for the center of DSC while the even number is for the positions between two DSCs. The position of DW is marked by the white (a), red (d), and black (e) dashed boxes, respectively.

1T‐TaS_2_ undergoes a series of temperature‐dependent phase transitions from an incommensurate CDW order to a commensurate CDW (CCDW) order, either for bulk crystals or ultrathin flakes.^[^ ^13^ ^]^ **Figure** **1**a presents a typical STM topography image of the low‐temperature CCDW phase in 1T‐TaS_2_ below the bulk transition temperature of 180 K. The protrusions correspond to the well‐known √13 × √13 CDW superlattice.^[^ ^21^ ^]^ Each CDW unitcell, so‐called a David‐star cluster (DSC) contains 13 Ta atoms (each with a single *d_z_* electron), where the 12 outer Ta atoms are displaced toward the central Ta atom (Figure S1, Supporting Information). This reconstruction leaves only a single unpaired *d_z_* electron in each unit cell, which is highly localized at the central Ta atom. In addition to regular CDW unit cells, a bright chain structure appears at the center of the topography. This kind of 1D structure has been well characterized as a CDW DW due to the translative phase shift of CDW between neighboring CCDW domains.^[^ ^18^, ^22^, ^23^ ^]^ This 1D DW is terminated by junctions with other types of DWs or kinks and we focus on the straight segment extending about 40 nm as shown partly in Figure 1a.

The tunneling dI/dV curves in Figure 1b are measured at different locations on the surface, which represent local density of electronic states (LDOS). The band gap of about 0.4 eV is observed in the CCDW domain (spot A in Figure 1a), while in‐gap states of the DW (spot C) and domain‐edge (DE) unit cells (spots B and D) manifest themselves at empty‐ and filled‐states, respectively. These spectroscopic features around the Fermi energy are consistent with the preceding STM studies on the CCDW domain and DWs.^[^ ^22^, ^23^ ^]^ In addition, a strong NDR effect is observed exclusively at the DE unit cells in both dI/dV (Figure 1b) and I/V (Figure 1c) at the sample bias of −126 mV (spot B) and −141 mV (spot D), which are just below the strong in‐gap state. Lateral variations of the relevant electronic states and the NDR feature are well captured in the line map of dI/dV crossing a DW as shown in Figure 1d,e. It is worth noting that the intensity of the NDR state for the left DE (spot B) is stronger than that of the right DE (spot D) at −130 mV in Figure 1d, which is consistent with those in Figure 1b,e. The NDR (dark blue contrast in the map) is well localized within a single DE unit cell on both sides of the DW. The on/off logical state of this NDR behavior is well‐controlled by varying the tunneling bias voltages (see also Figure S2, Supporting Information). In Figure 1e, the top of the valence band of the CCDW domain exhibits an upward band‐bending as approaching the DW. Such a local band‐bending near a DW is known to come from various factors such as distortion of David‐star clusters (DSCs) and charge transfer, depending on the type (structures and electronic states) of DW. In the present case, band‐bending is primarily a result of the Fermi‐level alignment between the domain and DW states. Within the DW, the CDW band gap is significantly reduced due to the imperfect DSCs and the *d*_z_ electrons form unoccupied in‐gap states so that the Fermi energy is located within a small band gap between this in‐gap state and the valence‐band edge. In order to align the Fermi levels of the CDW domain and the DW, the whole occupied bands should gradually move upward when approaching to the DW, which narrows the local band gap and induces a strong in‐gap state at the DE unit cell. That is, the in‐gap state at the DE comes from the state at the top of the valence band of the CCDW phase.

To understand the NDR behavior, it is crucial to understand the spectral feature at the top of the valence band as shown above. The previous works established that this feature is due to the unpaired *d_z_* electron localized at the central Ta atom of the DSC.^[^ ^20^ ^]^ In the single‐layer limit, this electron forms a half‐filled nearly flat band at the Fermi energy, which splits into a lower and an upper Hubbard band (a LHB and an UHB) through on‐site Coulomb repulsion. In the bulk with the bilayer stacking preferred, the unpaired *d_z_* electrons between neighboring layers pair to drive the system into a trivial band insulator with a bonding‐antibonding gap.^[^ ^24^ ^]^ At the surface of a bulk crystal, however, at least two different terminations are possible, namely the intra‐ and inter‐bilayer termination. The former has a trivial gap but the latter recovers a Mott gap.^[^ ^25^, ^26^ ^]^ These two different terminations have been distinguished by distinct STS dI/dV spectra. The dI/dV spectrum of the CCDW domain (curve A in Figure 1b) reveals a Mott‐insulating characteristic of the inter‐bilayer termination (so‐called C‐stacking) with UHB (LHB) at +0.2 eV (−0.2 eV).^[^ ^26^ ^]^ An extra filled‐state feature at −0.13 eV was attributed to the hybridized state of LHB with sublayers.^[^ ^24^ ^]^ At a lower energy of around −0.3 eV, the major valence band is located, which comes from the paired *d_z_* electrons within a DSC. The LHB at the DE unit cell is highly localized in space and energy due to its highly localized orbital within a DSC and the local band‐bending caused by the DW. This provides an ideal situation for NDR as detailed below.

### 2.2 Topography and Band Structure of 1D Channels

**Figure 2:**
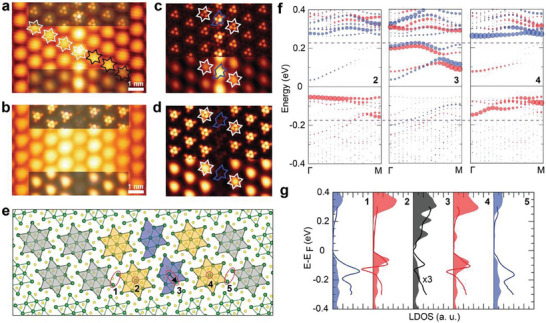
Structure of 1D NDR channels. a,b) STM topography images of the 1D channels under the scanning bias of +0.4 (a) and −0.13 V (b). Insets are the simulated STM images in (c,d). c,d) Simulated STM images at +0.4 and −0.15 V. The top panels show electron density at a constant height of 2.8 Å away from the top S atoms. The bottom panels display Gaussian‐blurred images with a standard deviation of 1.85 Å. e) Atomic structure of DW‐1. Blue, yellow, and gray colors represent the domain wall, domain edges, and domain regions. f) Band structure. The circle size is proportional to the localized states domain wall and domain edges marked in (e). g) Theoretical LDOS (filled curves) compared the experimental results (solid lines).

For a quantitative analysis of the NDR effect, however, we have to detail the atomic structure and electronic states of the DW and DE unit cells. **Figure** **2**a,b shows the empty‐ and filled‐state STM images of the DW, respectively. Although the atomic resolution image of unit cells is not achieved in the present work, the CDW phase shift can be unambiguously quantified (the black arrow in Figure 2e and Figure S3, Supporting Information), which indicates the existence of a DW.^[^ ^18^, ^22^, ^27^ ^]^ This phase shift corresponds to the first type of DW (so called DW‐1) among the twelve possible DW configurations (phase shifts) tabulated previously.^[^ ^18^, ^23^ ^]^ The fully relaxed atomic structure of DW‐1 obtained by DFT calculations is shown in Figure 2e. The DW‐1 structure consists of a single row of imperfect DSCs, each with 12 Ta atoms. In contrast, the DE unit cells have an almost ideal DSC structure. The simulated STM images based on this structure model reproduce the experimental images excellently as shown in Figure 2c,d. Note that this structure is different from what was observed and calculated for the same phase shift,^[^ ^23^, ^28^ ^]^ which has a double row of imperfect DSCs apparently. The previous theoretical work already showed that the single‐and the double‐row structures are almost degenerate in energy with a small energy difference of less than 5 meV/DSC. The brighter (dark) protrusions (depression) at DW in the empty (filled) state STM image are consistent with the emergence of in‐gap states at around 0.1–0.2 eV (Figure 2f) (the absence of the localized states at DW in the corresponding bias). The calculated electronic states of DW‐1 are in good agreement with the dI/dV spectra as shown in Figure 2g. The well‐localized 1D in‐gap states of DW in the empty states and the shifted LHB of DEs are well reproduced in the band structure (Figure 2f). The DW in‐gap state does not substantially hybridize with the electronic states of neighboring DE unit cells.

### 2.3 Intralayer Coupling Effect on NDR Behavior

**Figure 3:**
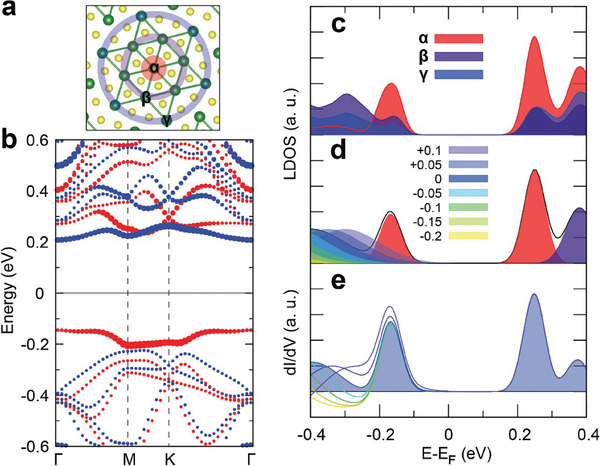
The role of intralayer coupling effect. a) Atomic structure of commensurate change–density‐wave phase. b) Band structure. Red and blue circles represent majority and minority spins, respectively. The circle size is proportional to the states localized at the central site Ta atom of the David‐star cluster. c) Local density of states obtained by DFT calculation. α, β, and γ denote the localized states at the center, middle, and outer Ta atoms marked in (a), respectively. d) Gaussian‐approximated LDOS as a function of the bulk‐level shift. e) Simulated dI/dV spectra using the WKB model.

Generally, a flat band characterized by a high density of state in a narrow energy range can induce a strong resonant tunnelling behavior, a greatly enhanced tunnelling current followed by a drastic drop as the voltage increase slightly, which generates NDR as observed in materials such as multilayer graphene and a few others.^[^ ^29^, ^30^, ^31^ ^]^ In a DSC of the CCDW phase, the nearly flat LHB exists at −0.17 eV due to the highly localized electron at the central Ta atom, whereas the major valence band is situated just below the LHB (**Figure** **3**b). The valence band electrons are distributed over the whole cluster as shown in the site‐resolved LDOS (Figure 3c) so that there exists finite the intralayer orbital overlap between the central (site α) and outer sites (β and γ) of a DSC. This orbital overlap may suppress the NDR effect in the CCDW phase. We simulate the orbital overlap with one narrow and the other wide Gaussian LDOS distributions for the LHB and the valence band (Figure 3d), respectively. The orbital overlap was controlled by shifting the energy center of the valence band LDOS and the tunneling current was calculated with the Wentzel–Kramers–Brillouin (WKB) approximation (details in Method).^[^ ^20^, ^32^ ^]^ Figure 3e displays simulated dI/dV spectra as a function of the valence‐band energy shift. One can confirm that the NDR effect due to the flat band is gradually suppressed as the orbital overlap increases. It is important to note that STS measurements usually involve averaging DOS over an area larger than a single atomic size. Therefore, the in‐plane orbital overlap in the STS spectra must be more significant than in the above calculation.

### 2.4 Origin of the 1D NDR Effect in the DW‐1 Configuration

**Figure 4:**
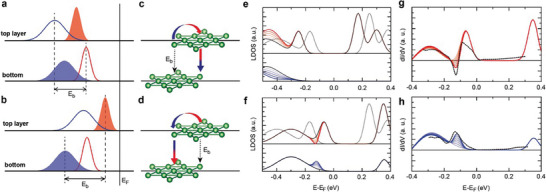
The interlayer charge transfer effect. a,b) Relative energy level of the central site between top and second layers at domain and domain edges, respectively. c,d) Schematic representation of the current flow between top and second layers at domain and domain edges, respectively. e,f) Gaussian‐approximated LDOS (top panel: central site and bottom panel: edge site) at domain and domain edges, respectively. Solid and dashed lines represent the LDOS of the top and second layer, respectively. Modified LDOS due to the current flows is indicated by the red and blue solid lines. g,h) Simulated dI/dV spectra using the WKB model (solid lines) compared to the experimental results (dashed lines) for the central and edge site, respectively.

Since the above intralayer orbital overlap exists also in the DE DSC, the occurrence of the strong NDR in DE requires an extra mechanism. We suggest that the interlayer tunneling plays a key role. **Figure** **4**a,b shows the relative energy levels between top and second layer for the domain and DE regions, respectively. Recent DFT calculations showed that the LHB state of the top layer in the CCDW domain in the inter‐bilayer termination is located at −0.21 eV, while the second layer's is at −0.14 eV (Figure S4, Supporting Information).^[^ ^24^ ^]^ Namely, the flat LHB (depicted by a red filled curve) of the top layer is lower than that of the second layer (red empty curve) in the domain region. In this case, the electrons at the central Ta site (α site in Figure 3a) of the top layer can have a higher possibility to tunnel into outer Ta atoms (γ cite, marked in Figure 3a) of the second layer. In such a case, the single‐layer LDOS (black lines in Figure 4e) can exhibit a tendency to increase (decrease) at α (γ) site, as shown in Figure 4e (see also Figure S5, Supporting Information, for details). In sharp contrast, the energy shift due to the band‐bending at the DE separates the LHB state of the top layer from the electronic state of the second layer to prevent direct tunneling into the site underneath. The tunneling into the second layer occurs via the intralayer hybridization path as shown in Figure 4d. With this, the LDOS of the central site is reduced while it is enhanced at the outer sites, as shown by the red and blue curves in Figure 4f (top and bottom, respectively). This contrasting interlayer electronic coupling of DW and DE unit cells results in contrasting I–V (dI/dV) behavior as simulated through the WKB approximation. Most notably, the simulation reproduces the strong NDR effect on the central site of the DE as shown in Figure 4g. The simulated dI/dV spectra in Figure 4g,h, obtained from the LDOS in Figure 4f, are in good agreement with the experimental dI/dV curves for the DE. The NDR state emerges at the central site of the DB, and the additional peak appears at the off‐center site at that energy. This aligns well with the experimental observation in Figure 1f, where off‐center peaks are observed at the energy corresponding to the appearance of the NDR state. The present mechanism of the NDR with the interplay of the intralayer and interlayer orbital overlap is consistent with that of the recent study for the 2D NDR at a high energy (0.8 eV) empty state for the CCDW phase of 1T‐TaS_2_.^[^ ^20^ ^]^

### 2.5 Tunneling Current‐Dependent NDR Behavior

**Figure 5:**
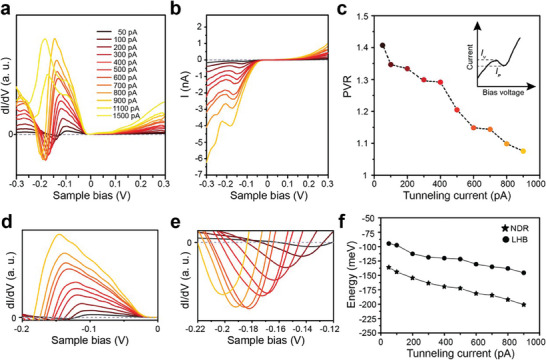
Tunable NDR behavior via changing tunning distance. a) A series of STS spectra taken on the left domain edge with the tunneling current ranging from 50 pA to 1.5 nA. b) Corresponding I–V curves taken at the same position in (a). c) Peak‐to‐valley ratio (PVR) versus Tunneling current. d,e) Highlighting LHB and NDR states dependence of tunneling current. f) Tunneling current‐dependent energy position of LHB and NDR states.

We further investigated how the NDR effect depends on the STM tunneling junction. We measured a series of dI/dV (I–V) curves at the DE with the tunneling current set point varied, as shown in **Figure** **5**a,b. The strength of the NDR, as measured by the peak‐to‐valley current ratio (PVR) linearly decreases as a function of tunneling current (Figure 5c). This decrease is accompanied by the increase of the LHB tunneling LDOS and, thus, indicates that less electrons from the LHB contribute to NDR. Note that the NDR disappears above the tunneling current of 1.0 nA. At the same time, the energy positions of the LHB state and NDR feature (zoomed up in Figure 5d,e) shift systematically; their energies (bias) shift linearly to a lower occupied state (Figure 5f). In principle, the very local tunneling current and electric field around the tunneling tip can alter the electron density and the energy level alignment within the intralayer unit cell and between the top and the second layer, which in turn change the NDR. The simulated result with the Gaussian broadening of the intralayer energy levels varied reasonably reproduces the observed electronic density of states under different tunneling distances (see Figure S6b, Supporting Information). However, the energy bands shift is not observed, and it also does not explain the decrease and the disappearance of the NDR feature in the high tunneling current regime. In our current tunneling junction, there exists a mismatch of work function between STM tip (PtIr) and 1T‐TaS_2_, which can give rise to the local electric field. We propose that this field would shift the LHB states of the top layer downward, bringing the energy level configuration closer to the case of the CCDW domain discussed above (Figure 4a), where the NDR is suppressed. Theoretically, once the LHB shift is taken into account in the model (see Figure S6c, Supporting Information), the more pronounced energy shift of LHB and NDR states together with the degeneration of NDR behavior would appear, which is in good agreement with the experimental data. The above results unambiguously show that the NDR effect in the present system is well controlled by tunneling condition or the local electric field.

## 3 Conclusion

We have uncovered a 1‐nm‐scale 1D NDR channel on the surface layer of a van der Waals system of 1T‐TaS_2_. The NDR channel is formed along the DW of the correlated CDW phase and is related to the lower Hubbard band of the Mott insulating state of this unusual CDW phase. NDR behavior based on a Mott state is unique. The intralayer and interlayer couplings of very well‐localized energy levels are crucial for the occurrence of the NDR states in this system. The local band‐bending near the DW to induce a 1D in‐gap state is important to form the 1D NDR channel. The present work contributes to a fundamental understanding of the emergence of NDR states in layered systems. On the other hand, the present finding of a well‐defined 1D NDR channel in a van der Waals layer with a nanometer‐scale dimension has strong merit for NDR device architecture based on 2D material systems.

## 4 Experimental Section

### STM/STS Measurements

1T‐TaS_2_ single crystal was grown by the chemical vapor transport technique. We cleaved the sample in the load‐lock chamber at room temperature under the pressure of 1 × 10^−9^ Torr to achieve a clean top surface. After the cleavage, the 1T‐TaS_2_ sample was transferred into the scanning chamber for STM and STS measurements in ultra‐high vacuum at *T* = 4.4 K. The mechanically sharpened PtIr wires were utilized as the STM tips. The differential conductance dI/dV curves were recorded using a standard lock‐in technique with a 10 mV bias modulation at a frequency of 1 kHz.

### DFT Simulations

Density functional theory (DFT) calculations were performed by the Vienna ab initio simulation package, implementing the projector augmented wave method and the Perdew–Burke–Ernzerhof functional.^[^ ^33^, ^34^, ^35^ ^]^ For electron‐correlation calculations, we incorporated an on‐site Coulomb energy of *U* = 2.3 eV.^[^ ^36^ ^]^ We employed a plane‐wave basis with a cutoff of 259 eV and Brillouin‐zone integrations of a 6 × 6 × 1 k‐point mesh for the √13 × √13 Brillouin‐zone. The single‐layer 1T‐TaS_2_ surface was modeled by a periodic slab with a vacuum spacing greater than 20 Å. We conducted atom relaxation until residual force components were within 0.02 eV. To generate simulated dI/dV curves, we utilized a standard I–V formular as 𝐼 ∝ ∫ 𝐷_𝑠_(𝐸) 𝐷_𝑡_(𝐸)𝑇(𝐸)[𝑓_𝑠_(𝐸) − 𝑓_𝑡_(𝐸)]𝑑𝐸,^[^ ^32^ ^]^ where D_s_ and D_t_ represent the density of states of the sample and tip, respectively. f_s_ and f_t_ are Fermi–Dirac occupation for the sample and tip, respectively. For the tunneling matrix of T, we applied the Wentzel–Kramers–Brillouin approximation, expressed as 𝑇 = 𝑒^−𝑧√∅−𝐸+𝑉/2^.^[^ ^20^ ^]^ Here, Ø represents the work function of the 1T‐TaS_2_ (4.96 eV). For simplicity, we set to the tunnel barrier width (z) to 1, corresponding to the tip‐sample distance. For the tip DOS, a Gaussian feature localized at the Fermi surface was modeled, based on previous theoretical models.^[^ ^30^ ^]^

## Conflict of Interest

The authors declare no conflict of interest.

## Supporting information



Supporting Information

## Data Availability

The data that support the findings of this study are available from the corresponding author upon reasonable request.
