# Supplementary material for: Nanometer‐Scale 1D Negative Differential Resistance Channels in Van Der Waals Layers
Source: Adv Sci (Weinh). 2024 Nov 13;12(2):2408090. doi: 10.1002/advs.202408090 (PMC11727404; doi:10.1002/advs.202408090)
Supplement: Supplementary file 1 — Supporting Information [file ADVS-12-2408090-s001.pdf]

ADVANCED SCIENCE
Open Access

# Supporting Information

for *Adv. Sci.*, DOI 10.1002/advs.202408090

Nanometer-Scale 1D Negative Differential Resistance Channels in Van Der Waals Layers

*Qirong Yao, Jae Whan Park, Choongjae Won, Sang-Wook Cheong and Han Woong Yeom**

Supporting Information

# Nanometer-scale one-dimensional negative differential resistance channels in van der Waals layers

*Qirong Yao, Jae Whan Park, Choongjae Won, Sang-Wook Cheong, and Han Woong Yeom**

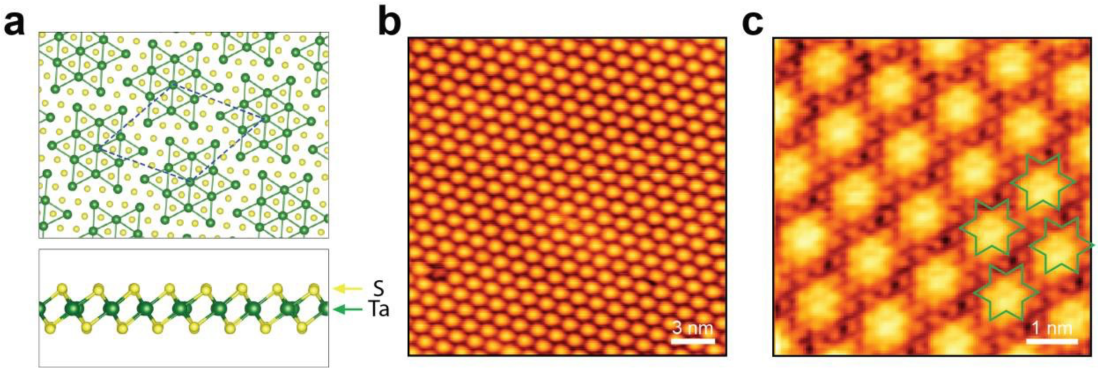


**Figure S1.** (a) Crystal structure models of 1T-$TaS_2$. (b) STM topography image of pristine 1T-$TaS_2$ surface with CCDW phase. (c) Atomically resolved David-star clusters on the top surface.

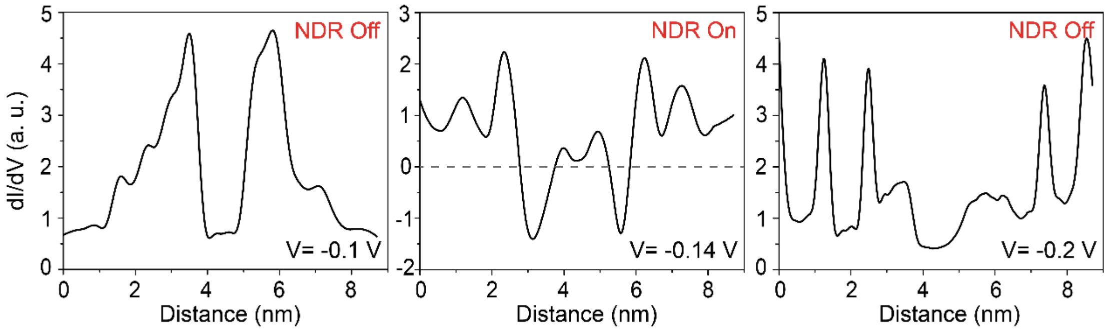


**Figure S2.** On/off logic states of the NDR behavior under a series of sample biases.

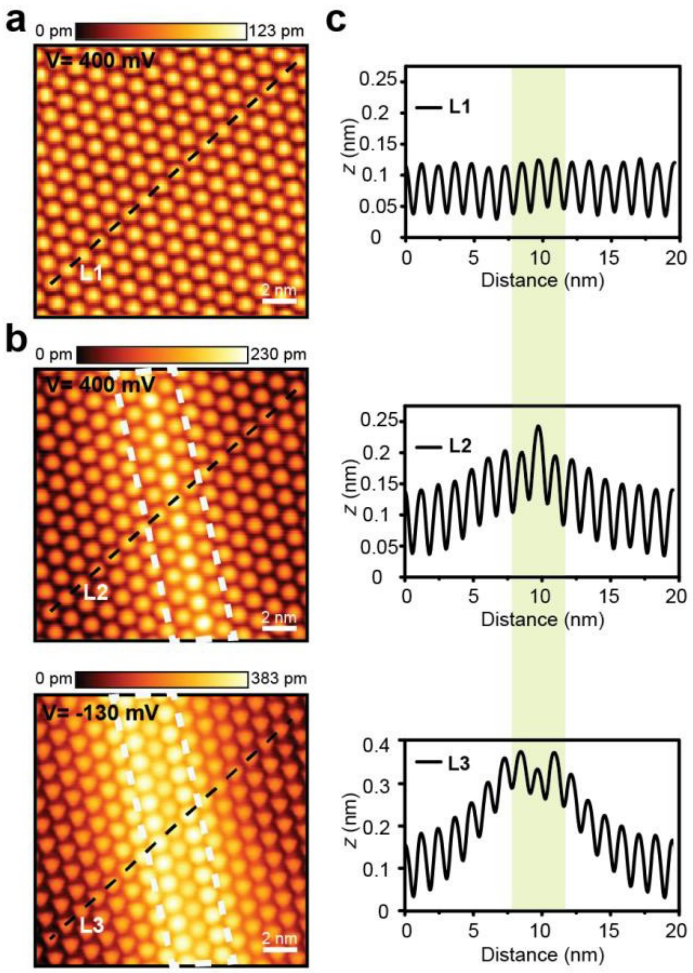


**Figure S3.** (a) STM topography image of the pristine 1T-$TaS_2$ surface under $V_{bias}$= 0.4 V. (b) STM topography images of the 1T-$TaS_2$ surface with 1D electronic channels taken at $V_{bias}$= 0.4 V and -0.13 V. (c) Height profile along the black dashed lines in (a & b), named L1, L2, and L3, respectively.

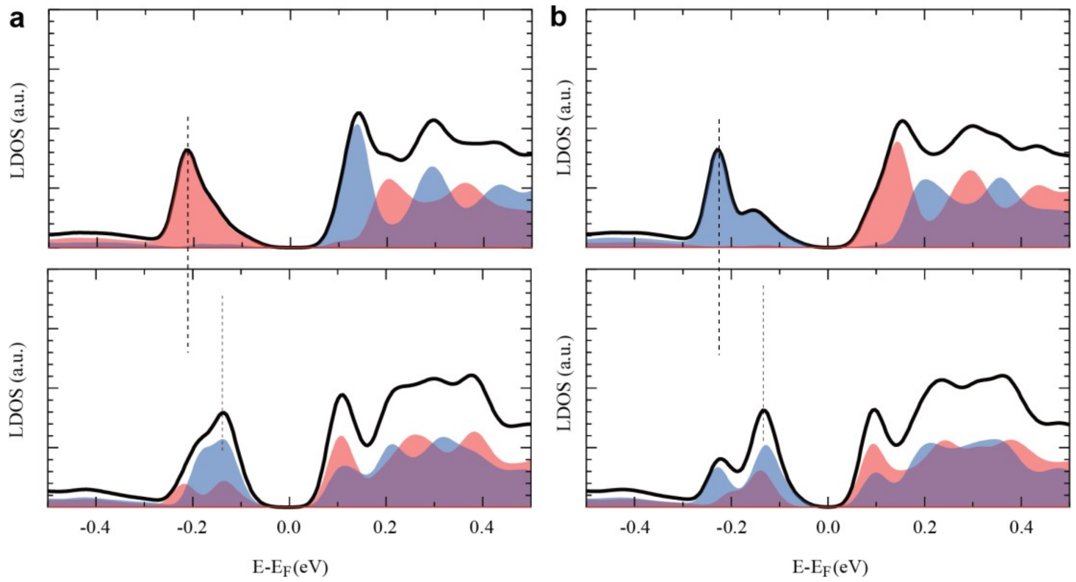


**Figure S4.** Theoretical LDOS of the C-stacked surface. (a) Antiferromagnetic order between top and second layers. (b) Ferromagnetic order. Top (bottom) panel represents the states localized at the top (second) layer. Black, red and blue curves correspond to total, majority and minority spins, respectively.

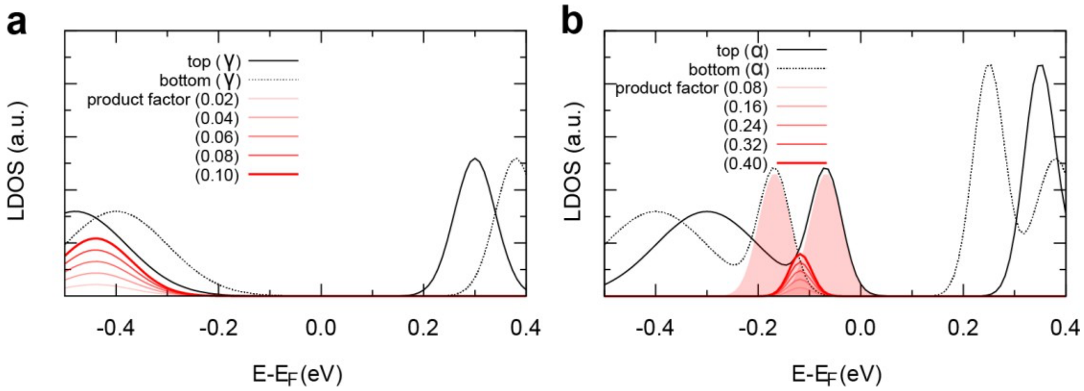


**Figure S5.** Estimation of LDOS variation due to current flow. (a) Domain region. Black solid line represents the LDOS of the top-layer CDW band (γ site) as the initial state for the current flow. Dashed line represents the LDOS of the bottom-layer CDW band as the final state. The current is proportional to the product of LDOS between initial and final state (red lines). (b) Domain edge. For the domain edges, current flows from top LHB state to the bottom LHB state as highlighted by red filled curves.

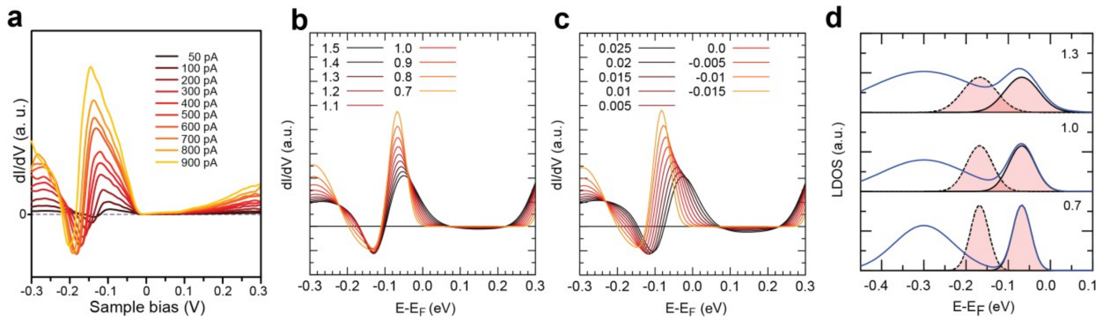


**Figure S6.** (a) A series of STS spectra. (b) Simulated dI/dV curves varying with the Gaussian broadening factor of c. In the ideal Gaussian LDOS, we employed σ1 = 0.031 for the localized state at the center and σ2 = 0.1 for the hybridized states with CCDW bands. These parameters are then adjusted as σ1' = cσ1 and σ2' = cσ2, respectively. (c) Same as (b) but the energy level of the LHB is shifted from 0.025 to -0.015 eV. (d) LDOS for three different c factors. Blue lines denote the top-layer LDOS and black solid and dashed lines highlight the localized states at the top and second layer, respectively.
